# Continuous theta burst stimulation in the treatment of epilepsia partialis continua: a case series

**DOI:** 10.1186/s42234-025-00175-8

**Published:** 2025-05-23

**Authors:** Chloé Algoet, Sofie Carrette, Alfred Meurs, Ann Mertens, Dimitri Hemelsoet, Paul Boon, Kristl Vonck

**Affiliations:** 1 Department of Neurology, Ghent University, Corneel Heymanslaan 10, Ghent, 9000 Belgium; 2 Department of Electrical Engineering, Eindhoven University of Technology, Eindhoven, the Netherlands

**Keywords:** Continuous theta burst stimulation, Repetitive transcranial magnetic stimulation, Epilepsia partialis continua, EPC, Status epilepticus, Neuromodulation

## Abstract

**Background:**

Epilepsia partialis continua (EPC) is a medication-resistant form of focal status epilepticus (SE), causing significant morbidity. This case series explored whether continuous theta burst stimulation (cTBS) could reduce seizure activity in patients with EPC.

**Methods:**

Three patients with motor EPC (2M/1F) underwent an accelerated cTBS protocol over four consecutive days (five 40-s trains/day, 5Hz bursts, 3 pulses at 50Hz/burst). Stimulation targeted the epileptogenic zone using a figure-of-eight coil at 80% of the resting motor threshold. Electroencephalography (EEG) was conducted before and after each session. Seizure frequency, intensity, adverse events (AEs), seizure diaries, and follow-up data were assessed.

**Results:**

cTBS did not interrupt EPC in any patient. One patient reported a 17% reduction in seizure frequency. Another noted mild improvement in shoulder jerks, and a third reported reduced arm tension, though without clinical confirmation. EEG showed no significant changes. One patient experienced seizures during stimulation, and another reported worsening of pre-existing headaches.

**Conclusion:**

In this small case series, a four-day accelerated cTBS protocol did not yield clinically meaningful seizure control in EPC. Further research is needed to evaluate TMS and TBS in SE and EPC, where a significant treatment gap remains.

## Introduction

Status epilepticus (SE) is defined as an abnormally prolonged seizure episode, arising from dysfunction of mechanisms responsible for seizure termination, spread or recurrence of seizures (Trinka et al. 2015; Lowenstein et al. 1998; Hanhan et al. 2001). Epilepsia partialis continua (EPC) is a specific subclass of focal motor status, marked by continuous epileptic fragments (motor or sensory), while consciousness remains intact (Trinka et al. 2015; Mameniškienė and Wolf 2017; Muthaffar and Alyazidi 2024). These episodes persist for ≥ 1 h and are associated with localized epileptic activity, typically causing seizures in a specific body part (Trinka et al. 2015; Mameniškienė and Wolf 2017; Muthaffar and Alyazidi 2024). One notable aspect of EPC is its heightened sensitivity to external stimuli, including sensory input and movement (Mameniškienė and Wolf 2017). Symptoms persist during sleep and are often accompanied by postictal weakness (Mameniškienė and Wolf 2017; Muthaffar and Alyazidi 2024; Khan et al. 2024). According to a recent European survey, the time course of EPC can be categorized into four distinct types: solitary episode, chronic repetitive non-progressive, chronic persistent non-progressive and chronic progressive (Mameniškienė and Wolf 2017).

Although formal epidemiological data are lacking, literature suggests EPC is rare, accounting for < 3% of all epilepsy cases (Mameniškienė and Wolf 2017; Muthaffar and Alyazidi 2024). In adults, the incidence appears to be slightly higher in males compared to females; however, no significant gender differences have been observed in children (Mameniškienė and Wolf 2017; Muthaffar and Alyazidi April 2024; Khan et al. 2024; Kravljanac et al. 2013). In pediatric cases, inflammatory and immune-mediated disorders are the primary etiologies, whereas in adults, structural abnormalities are the most common cause of EPC (30–60% of cases)(Muthaffar and Alyazidi April 2024; Kravljanac et al. 2013). Other potential causes include metabolic, genetic or unknown factors (Mameniškienė and Wolf 2017; Muthaffar and Alyazidi 2024).

The prognosis of EPC largely depends on the underlying cause, with better outcomes in reversible etiologies (Khan et al. 2024). In pediatric patients, prognosis appears to be worse than in adults, with approximately 64.7% experiencing neurological consequences and 15.7% resulting in fatal outcomes (Khan et al. 2024; Kravljanac et al. 2013). In this population, predictors for neurological consequences include interictal EEG abnormalities and periodic EEG patterns (Kravljanac et al. 2013). For lethal outcomes in children, predictors are the onset of EPC during the terminal phase of disease and the need for intensive therapy (Kravljanac et al. 2013).

The treatment of EPC is primarily guided by its underlying etiology, as the condition often proves resistant to pharmacotherapy (Mameniškienė and Wolf 2017). While benzodiazepines can effectively interrupt EPC, they are unsuitable for long-term therapy (Mameniškienė and Wolf 2017; Muthaffar and Alyazidi 2024). A European survey indicated that topiramate and levetiracetam are among the most effective options for continuous treatment in this patient population (Mameniškienė and Wolf 2017; Muthaffar and Alyazidi 2024). In cases of Rasmussen encephalitis, surgical isolation of the affected hemisphere remains the only curative option, with a success rate of up to 80% in younger patients (Mameniškienė and Wolf 2017; Muthaffar and Alyazidi 2024). Immunomodulatory therapy may be considered if antiseizure medications (ASMs) fail or if the clinical presentation suggests an underlying autoimmune condition (Muthaffar and Alyazidi 2024).

Given the resistance to pharmacotherapy, there is growing interest in alternative treatments for EPC. Transcranial magnetic stimulation (TMS) is a non-invasive neurostimulation technique that generates a strong magnetic field (± 2 Tesla) by passing a current through a coil of copper wire (Mattioli et al. 2024; Tsuboyama et al. 2020). Based on Faraday’s law of electromagnetic induction, application to the scalp induces a weak secondary current (50–150 V/m) in the brain, modulating neuronal excitability (Mattioli et al. 2024; Tsuboyama et al. 2020). TMS can be delivered in a repetitive format (rTMS), utilizing either low frequency (LF-rTMS, ≤ 1 Hz) or high frequency (HF-rTMS, ≥ 5 Hz) stimulation.

Theta burst stimulation (TBS) is a more recently introduced rTMS protocol, that involves delivering three bursts of pulses at a high frequency (50 Hz), with an interburst interval of 20 ms (5 Hz) (Chung et al. 2015; Cole et al. 2024). TBS offers significant advantages over standard rTMS, including shorter stimulation times (1–10 min. compared to 20–45 min.), lower stimulation intensity, and longer-lasting cortical effects (Chung et al. 2015; Liu et al. 2024). Moreover, TBS appears to demonstrate similar efficacy and safety compared to standard rTMS in treating depression in both children and adults (Chung et al. 2015; Liu et al. 2024).

Continuous TBS (cTBS) involves delivering either 300 pulses over 20 s or 600 pulses over 40 s without interruption (Chung et al. 2015; Huang et al. 2005). This protocol is believed to induce long-term depression (LTD), reducing cortical excitability for ~ 20 min. (300 pulses) or up to 1 h (600 pulses). Intermittent TBS (iTBS), on the other hand, delivers 30 pulses over 2 s, repeated every 10 s, with a total of 600 pulses (Chung et al. 2015). This protocol is associated with long-term potentiation (LTP) and enhances motor cortex excitability for at least 15 min. (Chung et al. 2015; Park JH 2022).

Overall, TBS is considered a safe neuromodulation technique. The estimated crude risk of mild adverse events (AEs) is approximately 1.1%, which is comparable to the risk associated with HF-rTMS protocols (Chung et al. 2015). Common side effects include headaches and dizziness (Chung et al. 2015). Although TBS theoretically carries a higher potential for seizure induction than rTMS, it can also be viewed as safer because it uses fewer pulses, has a shorter duration and operates at lower intensity levels (Chung et al. 2015). This is supported by literature reporting a low crude risk of seizure per session, estimated at just 0.02% (Chung et al. 2015).

In 2018, the U.S. Food and Drug Administration (FDA) approved iTBS targeting the dorsolateral prefrontal cortex as a treatment for therapy-resistant depression (Cole et al. 2024). However, no cTBS protocols have been approved to date (Cole et al. 2024). To the best of our knowledge, three sham-controlled trials have evaluated cTBS targeting either the epileptogenic focus or the cerebellum as a treatment for drug-resistant epilepsy (DRE) (Carrette et al. 2022; Gundogdu Celebi et al. 2023; Wang et al. 2025). While the initial findings are promising, further research is needed to assess efficacy and mechanisms. We recently performed a systematic review and found 17 patients with EPC who were treated with rTMS, with a cessation rate of 12/17 (70.6%) (Algoet et al. 2025). However, there are currently no published case reports specifically investigating the use of TBS in EPC patients. In this report, we present three patients with EPC treated with cTBS in order to observe its potential effectiveness and safety profile.

## Material and methods

### Participants

Three patients with EPC were recruited from Ghent University Hospital. The study was approved by the local ethics committee, and all participants provided written informed consent.

### Stimulation protocol

Accelerated cTBS was delivered using a figure-of-eight coil (MagVenture, Farum, Denmark), positioned over the epileptogenic focus at the motor cortex. The stimulation target was initially identified through prior presurgical evaluation incorporating clinical, radiological, and EEG data. However, for this study, targeting was further refined according to clinical presentation: when motor jerks were localized to the hand, the corresponding hand motor area was identified by eliciting motor responses with the TMS coil at the same location. Neuronavigation or source localization techniques were not used.

Stimulation intensity was set at 80% of resting motor threshold (rMT), which was determined individually prior to treatment. Each treatment session consisted of five trains of 600 pulses, administered over 40 s with an interstimulus interval (ISI) of 600 s. Pulses were delivered in 5 Hz bursts, each composed of three pulses at 50 Hz. This stimulation protocol was adapted from psychiatric therapeutic protocols. We hypothesized that applying these parameters in a cTBS rather than iTBS format could yield therapeutic benefits for seizure control in EPC. The protocol was applied once daily for four consecutive days.

Patients were instructed to maintain adequate sleep and avoid caffeine before sessions.

### Outcome measures

Electroencephalogram (EEG) was recorded for 15 min before and after each session to assess neurophysiological changes, as well as 24 h after the final session. Clinical outcomes included seizure frequency (number per day) and intensity (qualitatively assessed based on amplitude and duration of motor jerks), derived from seizure diaries. AEs were systematically recorded throughout treatment and follow-up to monitor the safety of the treatment. Long-term outcomes were assessed during post-treatment clinical visits.

## Results and discussion

### Patient 1

A 23-year-old male with intractable EPC since age 10 presented with increased seizure frequency and difficulty maintaining therapeutic ASM levels. At admission, the patient averaged 11 seizures/day during the prior week. His typical seizures involved left-sided jerking movements, primarily affecting his hand, but sometimes extending to his left leg and face, causing head deviation and impaired walking. Occasionally, seizures progressed to secondary generalized bilateral tonic–clonic seizures. In addition, the patient suffered from constant jerks on the left side of his body, predominantly in the arm, which worsened by voluntary movement. Previous interventions, including multiple subpial transections in the right frontodorsal, precentral and postcentral areas in 2004, as well as the placement of a vagus nerve stimulator (VNS) in 2006, were unsuccessful. Over the years, various ASMs had been tried without achieving seizure control (Table 1).

**Table 1:** Antiseizure medications tried prior to theta burst stimulation

Patient	Therapy	Maximum daily dose	Reason for reduction or discontinuation
1	Topiramate	600 mg	NR
	Carbamazepine	1000 mg	NA
	Clobazam	20 mg	NA
	Pregabalin	1200 mg	Fatigue
	Lacosamide	400 mg	Fatigue, dizziness
	Clonazepam	1 mg	NA
	Tiagabine	NR	NR
	Levetiracetam	NR	NR
	Phenobarbital	NR	NR
	Phenytoin	NR	NR
	Lamotrigine	NR	NR
	Valproate	NR	NR
	Prednisolone	NR	NR
	Intravenous immunoglobulins	NR	NR
2	Levetiracetam	4000 mg	NA
	Clonazepam	0.5 mg	NA
	Lamotrigine	275 mg	NR
	Valproate	1600 mg	Not effective
	Lacosamide	300 mg	NA
3	Levetiracetam	2000 mg	Aggression and agitation
	Carbamazepine	1600 mg	Hyponatremia and dizziness
	Clonazepam	5 mg	NA
	Phenobarbital	150 mg	Patient preference
	Pregabalin	525 mg	NA

*mg* milligrams, *pd* per day, *NR* Not reported, *NA* Not applicable

Upon admission, the patient was on a regimen of phenytoin, topiramate, carbamazepine, clonazepam, pregabalin, and perampanel. Clinical examination revealed first-degree nystagmus on leftward gaze and persistent left arm myoclonus. Multiple technical investigations were conducted (Table 2).

**Table 2:** Technical investigations prior to theta burst stimulation

Patient	Technical investigation	Result
1	3 T MRI	Previously documented subpial transections in the right cerebral hemisphere + mild progression of cerebral atrophy on the right side compared to prior MRI
	VEM	Continuous epileptic activity over the right hemisphere, with a central focus
	Invasive VEM* (subdural grid right centroparietal cortex)	Continuous epileptic activity over the right hemisphere, with a central focus
	iSPECT	Left mesiotemporal hyperperfusion
	MEG	Focal epilepsy originating from the right central cortex, with frequent propagation
2	1.5 T MRI	Extensive corticosubcortical damage with cortical laminar necrosis + widespread gliotic changes bilaterally (L > R) + cortical hemosiderin deposition in affected areas
	F-18 FDG-PET	Left medial frontoparietal laminar necrosis
	MR spectroscopy	Left medial frontoparietal laminar necrosis
	Full-body PET	No signs of lymphoma or malignancy
	Cerebral angiography	No features suggestive of vasculitis
	Brain biopsy	Several possible differential diagnoses, including infectious etiology, Rasmussen encephalitis, and subacute sclerosing panencephalitis
3	1.5 T MRI	Post-surgical changes after right frontal trepanation + right frontal anterior, Rolandic and parietal tissue loss, with associated cystic encephalomalacia
	VEM	Focus at the level of the right hemisphere. The nature of the seizures could be compatible with a temporal origin
	F-18 FDG-PET CT	Right prefrontal and insular hypometabolism
	Brain biopsy	Tissue with a low degree of gliosis and microglial activation, not compatible with Rasmussen encephalitis, cortical dysplasia or neural storage disease
	Invasive VEM	NR

*T* Tesla, *MRI* Magnetic resonance imaging, *VEM* Video-EEG monitoring, *iSPECT* Ictal single-photon emission computed tomography, *MEG* Magnetoencephalography, *L* Left,* R* Right, *FDG-PET* Fluorodeoxyglucose position emission tomography, *PET* Position emission tomography, *CT* Computed tomography, *NR* Not reported

^*^conducted before the subpial transections

During admission, the patient was treated with intravenous (IV) clonazepam, which reduced seizure activity to baseline levels. Afterwards, the clinical team proceeded with cTBS as a therapeutic intervention. In the event of a positive response, chronic cortical stimulation (CCS) would be considered as a long-term treatment option. The patient experienced a seizure during and following the first stimulation session. On the second day, two seizures occurred during stimulation; on the third day, two seizures were recorded between stimulation trains.

In the week preceding cTBS, a median of 11 seizures per day (range: 7–14) was recorded. Following cTBS, this number decreased to a median of 8 seizures per day (range: 5–11), representing a 17% seizure reduction (77 per week vs. 64 per week). However, this reduction was not considered clinically significant. EEG recordings showed no notable changes in epileptiform discharges.

### Patient 2

A 31-year-old male with intractable EPC since August 2014, likely due to seronegative Rasmussen encephalitis, presented clinical deterioration. His epilepsy was characterized by continuous right-sided jerking movements, primarily affecting the arm and shoulder. Previously, he experienced focal seizures involving jerks on the right side of the mouth and focal seizures with paresthesia in the left arm, both of which had resolved with lamotrigine. Despite various ASMs and immunomodulatory treatments, the continuous jerks had not improved (Table 1).

Upon admission, the patient was on a regimen of levetiracetam, lamotrigine, clonazepam, lacosamide, methylprednisolone, rituximab and acyclovir. Clinical examination revealed spastic right-sided hemiparesis with neglect, motor aphasia, and continuous jerking movements affecting the right hemisoma, predominantly the right arm. The jerking movements were presumed to be of epileptic origin rather than spasticity-related, based on their rhythmic and stereotyped nature and a history of focal seizures in this area. Multiple technical investigations were performed (Table 2).

The clinical team decided to administer cTBS. During one session, involuntary jerks were observed in the chin instead of the hand. The patient tolerated treatment well and experienced no AEs.

While the patient reported no changes, his partner observed a slight improvement in intermittent shoulder jerks. The 15-min EEG conducted after the first session was of poor recording quality, and no EEGs were performed following the second, third or fourth session. Notably, tizanidine was initiated during treatment. Regarding seizure reduction, cTBS was deemed ineffective.

### Patient 3

A 52-year-old female with intractable EPC since 9 months of age presented with inadequate seizure control. Her seizures were characterized by dystonic flexion of the left arm, occasionally accompanied by jerking movements in the left shoulder. Additionally, the patient experienced continuous jerking in the left hand, which worsened under stress. In the past, she once had jerking movements affecting the left eye and mouth, associated with eye deviation, linked to an infection. Although VNS implantation in 1994 resulted in some seizure frequency reduction, her epilepsy remained insufficiently controlled. Consequently, in November 2004, she underwent right frontodorsal and parietal topectomies, along with transpial resections of the pre- and postcentral gyrus, to address a congenital cortical malformation. In 2010, the VNS battery and electrode were replaced due to a lead breakage. Over the years, various ASMs had been ineffective (Table 1).

Upon admission, the patient was on a regimen of pregabalin, carbamazepine, and clonazepam. Clinical examination revealed left hemiparesis, swan-neck deformities of the left hand, slurred speech, saccadic eye movements, first-degree horizontal gaze nystagmus, and continuous jerking of the left hand. Multiple technical investigations were performed (Table 2).

The clinical team decided to initiate cTBS. The VNS was turned off during the magnetic TBS treatment and reactivated afterwards, with its functionality checked after each stimulation session. During treatment, the patient experienced an exacerbation of chronic headaches, effectively managed with 1 g paracetamol.

Subjectively, the patient experienced a reduction in the tension of her left arm up until two weeks after the final stimulation session, though this was not externally observed. The 15-min EEGs conducted before and after each session showed no significant changes regarding the right-sided slowing and sporadic epileptic discharges.

## Discussion

In this case series, a novel 4-day cTBS protocol failed to achieve a clinically meaningful reduction in seizure frequency. While one patient experienced a modest 17% decrease, two others reported subjective improvements.

Clinical and preclinical research highlights substantial individual variability in neuroplastic responses to TBS protocols. Indeed, only about 25% of individuals exhibit the expected outcomes, suggesting that both intrinsic and extrinsic factors influence responsiveness (Park JH 2022; Li et al. 2019). Individual characteristics such as age, gender, handedness, skull morphology, and skin properties contribute to this variability, along with external influences like sleep deprivation, fluctuating attention, hormonal fluctuations, and pre-session physical activity (Jannati et al. 2023; Guerra et al. 2020a). Genetic factors, including the brain-derived neurotrophic factor Val66Met polymorphism, the ApoE ε4 allele, and the catechol-O-methyltransferase Val158Met polymorphism, also play a role (Jannati et al. 2023; Guerra et al. 2020a). To mitigate variability, our protocol included measures such as ensuring adequate sleep and avoiding caffeine. Further strategies, such as genetic pre-screening, standardized scheduling to account for circadian rhythms, and age-adjusted stimulation parameters, may enhance consistency (Guerra et al. 2020b; Opie et al. 2017)*.*

The lack of efficacy contrasts with our previous findings from a systematic review on TMS in refractory SE, where conventional rTMS protocols showed more promising results, although publication bias likely influenced these outcomes (Algoet et al. 2025). Several factors may explain the absence of therapeutic response in our patients. A higher stimulation intensity, longer ISI, and extended treatment duration may be necessary for clinically meaningful effects. Low-intensity TBS often fails to elicit consistent neuroplastic changes, whereas higher intensities (90–120% of rMT) yield more robust effects (Jannati et al. 2023). Rodent studies suggest that spacing iTBS sessions at least 1 h apart enhances previously saturated LTP, whereas shorter intervals fail to elicit additional effects due to less synaptic recruitment (Kramár et al. 2012). Clinically, increasing TMS sessions improves response rates in treatment-resistant depression, and extended protocol durations in refractory and super-refractory SE correlate with prolonged seizure control (Algoet et al. 2025; Yip et al. 2017). These findings suggest that long-term treatments may be required to induce effects, and that extending the treatment duration for those who did not meet the initial response criteria could potentially have enhanced overall response rates.

Optimizing the targeting approach could further enhance efficacy. In this study, targeting was based on functional identification of the motor area using TMS. While this method is pragmatic in a clinical setting, it may lack the precision of imaging-guided approaches, especially when combined with neuronavigation. Source localization, using EEG or integrated EEG-MRI data, could provide additional benefits by identifying the epileptogenic zone with greater spatial accuracy, allowing for more precise targeting. Studies using different coil orientations – such as anterior–posterior versus latero-medial – indicate that latency variations in evoked potentials can predict whether an individual will respond to cTBS/iTBS or show an opposite effect (Hamada et al. 1991). These inter-individual differences may stem from variations in intracortical network recruitment. Furthermore, large cerebral lesions alter head conductivity and electrical field distributions, raising concerns about the validity of targeting assumptions derived from healthy subjects (Minjoli et al. 2017). Secondary macrostructural changes such as cortical atrophy may further influence the field patterns (Minjoli et al. 2017). However, Minjoli et al. found that TMS-induced electrical fields using a figure-of-eight coil remained largely unchanged in lesioned brains (Minjoli et al. 2017).

Homeostatic metaplasticity is a concept suggesting that the history of postsynaptic neuronal activity regulates both the magnitude and direction of stimulation-induced plasticity, maintaining synaptic modifications within a functional range (Mastroeni et al. 2013). This concept supports the use of priming stimulation protocols to optimize neuromodulatory outcomes. Studies demonstrate that excitatory iTBS priming enhances the inhibitory effects of subsequent cTBS on the motor cortex (Todd et al. 2009). Thus, incorporating iTBS priming into our protocol could have potentiated the inhibitory effects of cTBS. Experimental data highlight the N-methyl-D-aspartate receptor (NMDA-R) NR2 A/NR2B subunit ratio as a key regulator of metaplasticity, with a lower ratio favoring LTP over LTD (Murakami et al. 2012). In SE, selective NMDA-R trafficking and NR2B-subunit upregulation may predispose synapses to LTP, thereby counteracting the desired LTD effects of cTBS and diminishing its therapeutic efficacy (San-juan et al. 2019; Naylor 2013).

Finally, the influence of ASMs on treatment efficacy must be considered. While most research focuses on LTP, some ASMs, such as lamotrigine and NMDA-R antagonists, suppress LTD-like effects (Table 3)(Hamed 2020). One of our patients was taking lamotrigine, which may have influenced treatment outcomes. Given that epilepsy patients in neuromodulation studies often receive multiple ASMs, future research should clarify the impact on TMS-induced plasticity and explore strategies to minimize pharmacological confounds in neuromodulation trials.

**Table 3:** Effect of antiseizure medication on plasticity

ASM	Effect on plasticity
Levetiracetam	Significant suppression LTP-like plasticity in human M1
Carbamazepine	↓ facilitation
Gabapentin	↓ facilitation No effect on LTP-like MEP response
Lamotrigine	Decreased LTP-like MEP response Decreased LTD-like MEP response
Topiramate	↓ facilitation No effect on LTP-like MEP response
Ethosuximide	Reversed the LTP-like response
NMDA-R antagonist	Decreased LTP Decreased LTD
Diazepam	Trend-level on LTP-like MEP response

*ASM* Antiseizure medication, *LTP* Long-term potentiation, *M1* Primary motor cortex, *MEP* Motor-evoked potential, *NMDA-R* N-methyl-D-aspartate receptor, *LTD* Long-term depression

Our recent systematic review reported a cessation rate of 70.6% in EPC patients following the administration of rTMS (Algoet et al. 2025). Although TBS has shown efficacy comparable to conventional rTMS in disorders such as depression, this equivalence may not extend to SE. Given the small sample size in the present study, any assertion regarding the potential superiority of one stimulation protocol over another remains speculative. Robust conclusions will require further trials, which remain challenging in this complex patient population.

In this case series, one patient experienced seizures during and after stimulation. However, given the pre-treatment median of 11 seizures per day, it remains unclear whether this was treatment-related or coincidental. Overall, our findings suggest the preliminary safety of cTBS in patients with EPC, consistent with previous studies that employed single cTBS trains in individuals with idiopathic generalized epilepsy and more intensive cTBS protocols in patients with drug-resistant epilepsy (DRE)(Carrette et al. 2022; Koc et al. 2017; Gundogdu Celebi et al. 2023).

## Conclusion

This is the first published case series investigating cTBS as a treatment for EPC. While our findings do not support a clinically meaningful seizure-reducing effect, they suggest preliminary safety. We advocate for further exploration of this approach following protocol optimization, including increased stimulation intensity, extended ISI, prolonged treatment duration for non-responders, refined target localization via neuronavigation and source imaging, optimized coil orientation, and incorporated priming techniques. Furthermore, structural abnormalities may alter the induced electrical field pattern, and ASMs can influence cortical excitability and plasticity. The substantial inter-individual variability in response to TBS challenges the feasibility of a one-size-fits-all approach, underscoring the need for personalized treatment protocols. Further research should focus on optimizing stimulation parameters, integrating predictive biomarkers, and establishing clear patient selection criteria to enhance the efficacy of cTBS in EPC.

## Data Availability

No datasets were generated or analysed during the current study.

## Abbreviations

AEs: Adverse events
ASMs: Antiseizure medications
CCS: Chronic cortical stimulation
cTBS: Continuous theta burst stimulation
DRE: Drug-resistant epilepsy
EEG(s): Electroencephalogram(s)
EPC: Epilepsia partialis continua
FDA: Food and Drug Administration
GABA: Gamma-aminobutyric acid
GABA-A-R: Gamma-aminobutyric acid receptor
HF-rTMS: High-frequency repetitive transcranial magnetic stimulation
ISI: Interstimulus interval
iTBS: Intermittent theta burst stimulation
IV: Intravenous
LF-rTMS: Low-frequency repetitive transcranial magnetic stimulation
LTD: Long-term depression
LTP: Long-term potentiation
NMDAR: N-methyl-D-aspartate receptor
rMT: Resting motor threshold
rTMS: Repetitive transcranial magnetic stimulation
SE: Status epilepticus
TBS: Theta burst stimulation
TMS: Transcranial magnetic stimulation
VNS: Vagal nerve stimulation
